# Quality of health care in Primary Care: perspective of people with Diabetes *Mellitus*

**DOI:** 10.1590/0034-7167-2023-0008

**Published:** 2023-10-06

**Authors:** Rosilene Rocha Palasson, Elisabete Pimenta Araújo Paz, Gerson Luiz Marinho, Luiz Felipe da Silva Pinto, Elen Ferraz Teston, Margareth de Almeida Gomes, Maria Helena do Nascimento Souza, Sonia Silva Marcon

**Affiliations:** I Universidade Federal de Mato Grosso do Sul. Campo Grande, Mato Grosso do Sul, Brazil; II Universidade Federal do Rio de Janeiro. Rio de Janeiro, Rio de Janeiro, Brazil; III Universidade Estadual de Maringá. Maringá, Paraná, Brazil

**Keywords:** Primary Health Care, Diabetes *Mellitus* Type 2, Quality of Health Care, Self Care, Patient Safety, Atención Primaria de Salud, Diabetes *Mellitus* Tipo 2, Calidad de la Atención de Salud, Autocuidado, Seguridad del Paciente, Atenção Primária à, Saúde, Diabetes *Mellitus* Tipo 2, Qualidade da Assistência à, Saúde, Autocuidado, Segurança do Paciente

## Abstract

**Objectives:**

to identify how people with diabetes assess the care offered by Primary Care teams.

**Methods:**

a cross-sectional study based on structured interviews with the application of the Patient Assessment of Chronic Illness instrument to people with Type 2 Diabetes *Mellitus*. Data were submitted to statistical analysis.

**Results:**

451 individuals participated in the study, more than half aged 60 years or older (64.0%); 63.9% had been diagnosed for more than five years; and 23.9% used insulin. The average score obtained was 2.5, which indicated little involvement in self-care and low support for the care of the chronic condition by the Family Health Strategy team, and was higher among women and people with a partner.

**Conclusions:**

people with diabetes consider that they do not receive individualized treatment, with dialogue and discussion for setting goals, and that they are not prepared for self-managing their health condition.

## RESUMO

**Objetivos:**

identificar como pessoas com diabetes avaliam a assistência ofertada pelas equipes da Atenção Primária.

**Métodos:**

estudo seccional, realizado a partir de entrevistas estruturadas com aplicação do instrumento *Patient Assessment of Chronic Illness* a pessoas com Diabetes *Mellitus* 2. Os dados foram submetidos à análise estatística.

**Resultados:**

participaram do estudo 451 indivíduos, sendo mais da metade com 60 anos ou mais (64,0%); 63,9% tinham diagnóstico há mais de cinco anos; e 23,9% faziam uso de insulina. O escore médio obtido foi de 2,5, o que indicou pouco envolvimento no autocuidado e baixo suporte ao cuidado da condição crônica por parte da equipe da Estratégia Saúde da Família, e foi maior entre as mulheres e pessoas com companheiro(a).

**Conclusões:**

as pessoas com diabetes consideram que não recebem tratamento individualizado, com diálogo e discussão para o estabelecimento de metas, e que não são preparados para a autogestão da condição de saúde.

## RESUMEN

**Objetivos:**

identificar cómo las personas con diabetes evalúan la asistencia ofrecida por los equipos de Atención Primaria. Métodos: estudio transversal, basado en entrevistas estructuradas con aplicación del instrumento *Patient Assessment of Chronic Illness* a personas con Diabetes *Mellitus* 2. Los datos fueron sometidos a análisis estadístico.

**Resultados:**

participaron del estudio 451 individuos, más de la mitad con 60 años o más (64,0%); el 63,9% tenía más de cinco años de diagnóstico; y el 23,9% usaba insulina. El puntaje promedio obtenido fue de 2,5, lo que indica poca participación en el autocuidado y bajo apoyo para el cuidado de la condición crónica por parte del equipo de la Estrategia de Salud de la Familia, siendo mayor entre las mujeres y las personas con pareja.

**Conclusiones:**

las personas con diabetes consideran que no reciben un tratamiento individualizado, con diálogo y discusión para el establecimiento de metas, y que no están preparadas para el automanejo de la condición de salud.

## INTRODUCTION

Within the scope of Primary Health Care (PHC), the care offered to people with chronic conditions has been progressively related to issues involving patient safety^(1-4)^. As actions are proposed for safe, effective, equitable, timely, efficient and person-centered care, practices that disseminate a culture of patient safety promote greater quality of care^(1)^. However, the processes that involve the implementation of these practices are complex and full of challenges, which are amplified when it comes to the care for people with chronic conditions^(1,4)^.

Several developing countries, including Brazil, are going through a moment of epidemiological transition characterized by high occurrences of chronic diseases, which represent the main causes of hospitalizations and deaths, especially among older age groups^(2)^. Cases of Diabetes *Mellitus* (DM) have increased considerably worldwide over the years, and in Brazil, between 2013 and 2019, there was a 24% increase in the prevalence of this condition^(5)^. In the last two decades, Rio de Janeiro was the capital of Brazil with the highest DM mortality rates (with an average rate of 40.4 deaths per 100,000 inhabitants).

In the Brazilian context, the provision of PHC for people with chronic conditions remains centered on medical consultations, which, in general, are short in duration and emphasize the prescription of medications. Moreover, the appreciation of different social realities and the integration of family and friends in the creation of bonds with the health unit and professionals are considered incipient, expanding the list of obstacles and challenges to the achievement of attributes inherent to the primary care model adopted in Brazil^(2-3)^.

In this regard, it is necessary to give visibility to the gaps that limit the safety culture in PHC, especially with regard to the collaboration, cooperation and involvement of people in their care as well as damage measurement and reporting^(4)^. Patient-centered care favors the development of self-care actions that, in turn, are relevant for the proper management of chronic conditions^(6)^.

Improving skills related to self-monitoring, identifying changes in functionality, managing symptoms and complications require a unique assessment and definition of goals with the co-participation of those involved. In this way, the encouragement of shared decision-making, based on empathy, autonomy, respect for patients’ choices and decisions, is associated with a reflective, motivating and creative practice, and requires that support for users is available whenever they have doubts or need to improve their performance^(7)^.

These provisions are part of the Chronic Care Model (CCM) set of interventions, which aims to improve the quality and management of chronic conditions^(8)^, through comprehensive changes in health systems, which involve culture, organization and effectiveness mechanisms for safe and quality care.

Considering these aspects, it is essential to know the perspective of people with a chronic condition in relation to the quality of health services. In this regard, studies have been carried out with the purpose of measuring the coherence between the care provided and CCM assumptions using the Patient Assessment Chronic Illness Care (PACIC)^(8)^. This instrument has already been applied to people with chronic conditions, such as DM^(9)^, metabolic syndrome^(10)^, hypertension^(11)^, depression^(12)^, multiple sclerosis^(13)^, osteoarthritis^(14)^, among others. It should be noted that, in the international context, the instrument was translated and validated in some countries, such as Germany, Denmark, Finland, Holland, France, Italy, Egypt, Spain, China, Japan, Saudi Arabia, Thailand, Korea and Vietnam, and observed there is a growing increase in publications on the subject^(15)^.

## OBJECTIVES

To identify how people with 2DM assess the care offered by Primary Care teams.

## METHODS

### Ethical aspects

The research protocol, originated from a doctoral thesis, was appreciated and approved by the Research Ethics Committee of the *Escola de Enfermagem Anna Nery* and *Instituto de Atenção à Saúde São Francisco de Assis, Universidade Federal do Rio de Janeiro* (EEAN-HESFA/UFRJ) and by the Research Ethics Committee of the Municipal Health Department (MHD/RJ), a co-participant institution in this study. After acceptance, all participants signed the Informed Consent Form, in two copies, of equal content.

### Study design, site, and period

The data analyzed in this study come from a doctoral thesis. This is a quantitative cross-sectional study, whose estimates sought to represent the population of adults enrolled in PHC units in a region of the city of Rio de Janeiro, with a medical diagnosis of DM. The STrengthening the Reporting of OBservational studies in Epidemiology (STROBE) recommendations, a tool that suggests standardization for carrying out cross-sectional epidemiological studies, guided the preparation of its report^(16)^.

The study was carried out in Primary Care health units, located in neighborhoods in the southern part of the city of Rio de Janeiro. It is a region that presents population profiles from different socioeconomic strata, where condominiums with “the most expensive square meter in the country” are a few kilometers from homes in extremely vulnerable conditions, thus delimiting a geographic space whose population profile is close to that observed for the municipality as a whole.

In 2013, approximately 40% of households in the city of Rio de Janeiro were registered in the Family Health Strategy (FHS), and, in 2019, the volume of registrations reached approximately 63% of households, indicating an annual increase of 31% and representing the highest growth among all capitals in Brazil^(17)^.

### Population or sample; inclusion and exclusion criteria

Adults with a medical diagnosis of type 2 Diabetes *Mellitus* (2DM) participated in the study, accompanied by FHS teams working in PHC units in the municipality.

The calculation of the investigated sample size considered the prevalence of 2DM of 8.2% for men and 11.2% for women^(18)^. Admitting a maximum error of 5% for the estimates and considering an increase of 5% for possible losses, a sample of 451 adult individuals was obtained. Study participants were selected from the lists of registered participants, according to sex and age group.

People aged 18 years or older and diagnosed with 2DM registered by FHS teams were included in the study. In turn, those with a language disorder and whose respondent would be a third party were excluded. The drawn people were initially contacted by telephone, and they were scheduled to attend the Basic Health Unit (BHU) for a consultation, according to their availability on the day and time. Up to three scheduling attempts were made, only to be replaced later by another person from the same FHS team with the same gender and age group.

### Study protocol

Data were collected by the main researcher, through individual structured interviews, carried out in a reserved room at the health unit, at home or in social facilities in the area covered with the application of PACIC.

The PACIC was developed by researchers and managers at the MacColl Institute for Healthcare Innovation, in Seattle, United States, to assess the quality of care consistent with CCM interventions^(8)^. The version, adapted for Portuguese, is composed of 20 items, divided into five dimensions: *adesão ao tratamento* (patient activation) (items 1-3); *modelo de atenção, apoio à tomada de decisão* (delivery system design/decision support) (items 4-6); *definição de metas e/ou adaptação* (goal setting) (items 7-11); *resolução de problemas/contextualização do aconselhamento* (problem-solving/contextual counseling) (items 12-15); and *coordenação da atenção/acompanhamento* (follow-up/coordination) (items 16-20)^(19)^.

Responses to the instrument are presented on a five-point Likert-type scale (almost never, generally not, sometimes, most of the time, almost always). The interpretation of results can be made from the total average score per dimension and per item. In all cases, a score above 3.0 indicates greater involvement/participation in self-care and support for the chronic condition care^(19)^.

It should be noted that, although the PACIC has already been used in numerous studies and in different countries, research carried out in Germany pointed out that the number of points on the response scale can affect the score obtained and compromise the interpretation of results^(12)^.

### Analysis of results, and statistics

Data were presented with summary statistics (mean, median and standard deviation), and comparisons were performed based on the ANOVA test to verify the association between PACIC scores and sociodemographic characteristics. The measure of association used was the Odds Ratio, with a respective Confidence Interval of 95% and a significance level of less than 5% (p < 0.05). Data were tabulated in Microsoft Office Excel^®^ and analyzed in the Statistical Package for the Social Sciences^®^ 20.0.

## RESULTS

The study included 451 people with a mean age of 63 years (standard deviation (SD) ± 11.1), 51.2% of whom were female. Thus, 59.1% declared brown color or race; 53.4% lived with a partner or spouse; and 81.8% had an income between one and three minimum wages. Most participants had been diagnosed with DM for more than five years (63.9%), and approximately ¼ used insulin (23.1%).

With a general average equal to 2.5 (SD ± 1.2), the indicator that assessed patients’ involvement with health services in the chronic condition (DM) management indicated a deficit in relation to self-care and low support from the health teams that follow them up. The PACIC dimensions that suggested better results were: “B) Delivery system design/decision support” (mean score = 3.3; SD ± 4.5); “C) Goal setting” (2.7; SD ± 1.2); and “D) Problem-solving/contextual counseling” (2.7; SD ± 2.7). The lowest averages were in the dimensions “A) Patient activation” and “E) Coordination of care/monitoring” (2.0; SD ± 1.8 and 1.9; SD ± 2.0, respectively).

**Figure 1:**
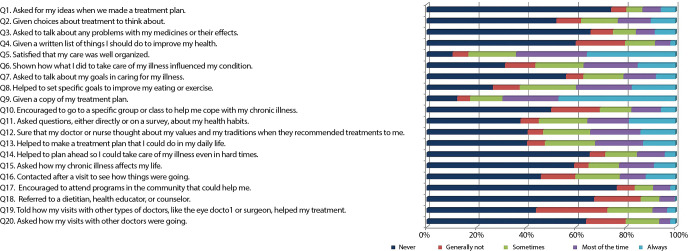
Distribution of responses to the Patient Assessment of Chronic Illness Care 20 items, Rio de Janeiro, Rio de Janeiro, Brazil

Considering the assessment of the answers to each item of the questionnaire, more than half of interviewees said “never” for 10 of the 20 items, with a higher concentration of “almost never” and “generally not” in the “patient activation” and “coordination of care/ monitoring” dimensions. In the “patient activation” dimension, it is noteworthy that 73.4% of participants reported that they were never asked for effective collaboration when defining their care plan; 51.7% stated that they were never given treatment options to think about; and 65.2% answered that the team does not ask about problems in medication use (Figure 1).

The “problem-solving/contextual counseling” subscale had the highest score (3.3), with the question about satisfaction with the treatment organization contributing the most to this result, since more than half of participants reported being “most of the time” (27.9%) or “almost always” (34.8%) satisfied, although 59.9% of them stated that they “almost never” received a list of “things” to improve their health.

As for the “setting goals” dimension, the score obtained was higher than the general score (2.7). Contributing to this result was the fact that almost 80% of participants stated that they “most of the time” or “almost always” received written information about their care plan. It is important to highlight that, in this case, they were referring to medication prescription and not to aspects related to changing behavior as part of treatment. Furthermore, 55.7% of respondents stated that they were “almost never” approached by the team about what they intended to do to improve their health conditions (Figure 1).

The “problem-solving/contextual counseling” dimension also obtained a higher score than the general one (2.7), although 65% of interviewees stated that they “almost never” received help to plan health care in difficult times and 56.2 % “almost never” were asked how the chronic condition affects their life (Figure 1).

Finally, the “follow-up/coordination” dimension obtained the lowest score (1.9), contributing to this, the fact that 75.8% of participants reported that they “almost never” received an incentive to participate in community programs; 67.2% “almost never” received guidance on DM care from other professionals who make up the multidisciplinary team (nutritionist, pharmacist, physical educator or nutritionist); 63.2%, that in general there are no questions about consultations with ophthalmologists and cardiologists; and 43.7%, that “almost never” were referred to specialists.

In the bivariate analyses, it was observed that, compared to women, men were twice as likely to be satisfied with organization of treatment (OR = 2.34; p<0.01) and to have contact with someone from the team after consultation (OR = 2.27; p<0.01). Women, in turn, had a 40.0% greater chance of participating in food reeducation groups compared to men (OR = 1.43; p < 0.01).

Furthermore, compared to participants who did not have a spouse or partner, those who reported living with a partner were more likely to adhere to the treatment offered by the family health team, since, in at least one item of each dimension of the scale, they reached scores that indicated greater participation in DM treatment and follow-up (p < 0.01).

## DISCUSSION

The present study identified that the health care provided by PHC team professionals, from the perspective of people with DM, rarely encourages their participation in the decision-making process that helps them to carry out self-care. This occurs because the study identified that FHS teams promote various actions to improve the quality of life of people with DM. Among the actions pointed out, mention was made of nutritional support groups, walks, people with diabetes and hypertension and health education, nutritional consultation, nursing, medical, distribution of medicines, home visits, among others, which corroborates findings identified in the study carried out in a medium-sized municipality in southern Brazil^(20)^.

Despite the variety of actions aimed at a better quality of life, they are not enough to guarantee care that favors safety and individuality in treatment, when presented to participants. The need for professionals to recognize users’ role in relation to their health condition and involvement in care planning actions is highlighted^(21)^.

The results suggest that, in the follow-up carried out by the teams under study, there is no individualized treatment, with dialogue and discussion, to establish priorities and goals together, evidencing that teams’ performance in relation to this chronic condition remains centered on antidiabetic prescription supply and on the vertical transmission of guidelines, with a generalized approach to changes in behavior. As a result, users may find it difficult to recognize themselves as participants in their care, which implies that the service does not favor activation and preparation for self-management of the health condition, which involves, for instance, improving skills, such as self-monitoring, management of symptoms and acute complications.

In the present study, the average score obtained in the PACIC indicated little involvement of individuals in carrying out self-care actions and low support from professionals, which corroborates the results found in a study carried out in two health districts in Minas Gerais (whose score was 1.5), which pointed out difficulties in the dimensions of teams’ proactivity and person-centered care^(3)^. It should be noted that interventions aimed at the centered care model demonstrate an increase in people’s involvement with self-care activities, with reports of greater personal control, awareness and coherent understanding of the condition. However, for this to happen, whether it is necessary to replace the usual topic verification approach with a more complex educational approach, in order to enable persons to be empowered to make decisions and manage their own health^(22-23)^.

Thus, encouraging shared decision-making based on empathy, respect for patients’ choices and decisions, is associated with autonomy: a reflective, motivating and creative practice that requires support for users to be available whenever they have doubts or need to improve their performance^(21)^. Faced with this, education for self-management is an intervention that promotes behavior change, characterized by a greater capacity of individuals to make decisions, when necessary, with a view to minimizing complications and maximizing their health condition. Most of the time, and for the rest of their lives, people with DM will live with situations in which they need to determine actions for their own well-being and, therefore, need to understand how to carry them out and be co-responsible for their care^(7)^.

A meta-analysis study pointed out that face-to-face or remote supervision actions and home visits are effective strategies in encouraging self-management of the health condition. Therefore, these need to be personalized, centered on people, and favor individuals’ ability to cope with their health, in addition to contributing to their safety^(23)^. In this way, PHC professionals can use methodological strategies and tools that provide case management, a motivational approach, or other possibilities, taking into account that people with DM should be motivated to improve treatment engagement and develop behavioral changes that enable better health care^(24)^.

Difficulties in providing a care approach that recognizes the essential role of users in managing their own health condition can have several explanations, such as the work process of professionals working in PHC. If one considers the quantitative ratio of people under the responsibility of a single team, it is not difficult to infer that high numbers of users overwhelm professionals and result in work overload, which may influence the operationalization of care, which limits the development of a dialogic approach.

Corroborating this idea, a study carried out with people with 2DM in Malaysia, which also used the PACIC to compare the quality of care in the presence or absence of professionals specialized in PHC, pointed out that the disproportion between the number of teams that had the presence of these professionals and the coverage area population was above the international standard. This condition was associated with lower quality perceived by users as well as with increasing age and the number of professionals available at the clinics^(25)^.

The literature points to other problems experienced by FHS teams that interfere with the provision of quality care to people with chronic conditions within the scope of PHC. Among them, mention is made of training, health professionals’ qualification and updating, the focus on pathology and physical care, influencing the care that is provided and a work process, and the organization of services aimed at the care of acute conditions^(3,26)^. In view of this, it is necessary that, during health professionals’ training, there is a concern to make them capable of carrying out interventions in line with the CCM, as professionals need to have skills for problem-solving and decision support, using shared decision-making and goal-setting to increase patient activation^(26-27)^.

Another aspect that draws attention in the results of this study was the low score obtained in the “follow-up/coordination” subdimension, characterized by the almost total absence of follow-up with other professionals, such as nutritionists, physical educators, or social workers. It is likely that this finding is related to the insufficient number of these professionals in the network, but also to the changes that occurred due to the change in the execution of the actions of these teams, since the period of data collection coincided with change in management.

A PACIC validity study, developed in the countryside of São Paulo, Brazil, with 85 people with DM, identified weakness in this subdimension. This was related to the difficulty in working in a multidisciplinary team, specifically with diabetes education, resulting in the detriment of comprehensive care^(28)^.

In the municipality of Rio de Janeiro, FHS teams had the support of professionals from the Expanded Family Health Support Center (NASF - *Núcleo Ampliado de Apoio à Saúde da Família*) for health care for the enrolled population. It is noteworthy that, despite variations in the work process, the main proposition of NASF is to favor the comprehensiveness and longitudinality of care, through actions that include co-management and matrix support between FHS and NASF teams, shared consultations and educational and/or therapeutic groups^(29)^.

However, in the present study, NASF professionals’ work was limited, since more than half of the interviewees reported not having been invited to participate in collective group activities on issues related to treatment, and this is in line with the findings of other studies^(30-31)^. It should be noted that NASF professionals’ work has the potential to promote the autonomy of users, as it favors health awareness and changes in lifestyle, especially in the case of people with chronic conditions. This care is possible based on the multidimensional assessment, which is performed by a multidisciplinary team in a collaborative way^(24,27)^.

The absence or deficiency of follow-up to verify vital organs’ conditions, through annual assessments with an ophthalmologist and a cardiologist identified in this same subscale, represents a care gap, since the possibility of chronic complications is frequent among people with DM. It should be remembered that PHC is recognized as the coordinator of users’ therapeutic path in the health system, aiming to reduce the fragmentation of care and in search of the effectiveness of comprehensiveness^(32)^.

According to research that analyzed PHC care coordination using data from the national program to improve the quality of access to primary care, in recent years there has been an evolution in the assessment of care coordination, but this is seen with apprehension due to changes in the national primary care policy that took place in 2017^(32)^. It is important to maintain the investment in components that favor the coordination of care in the health service network, and this involves clinical information systems’ availability, with electronic medical records, internet access, computers and telephones in BHU.

Furthermore, the limitations in communication between PHC and specialized care result in disagreements about inadequate treatment and referrals, which makes it difficult to articulate the Health Care Network with solutions, which may lead to fragmentation of care. This reproduces duplication, overuse of procedures, increased costs at all levels of care, in addition to the possibility of conflicting therapeutic plans, which does not benefit users^(26,33)^.

The findings of the research carried out in primary care services in Saudi Arabia, with people with DM and hypertension, using the PACIC, also identified low scores in the follow-up and coordination sub-dimension, suggesting the need for more referrals to specialists, carrying out follow-up via visits or telephone contact. It is noteworthy that, although there are differences in health systems in terms of funding and organizational policies, comparisons of data found in different countries can provide opportunities to learn from each other in terms of strengths and limitations of approaches to non-communicable diseases^(11)^.

In the present study, men were more satisfied with the quality of care, and reported greater attention from the team in visits after consultations, when compared to women. Another difference identified was the greater perception among women of incentives to participate in educational groups, which can be explained by the greater frequency of women in health services. A study carried out with men in northeastern Brazil identified the lack of knowledge of the services offered in PHC and the difficulty in performing quality self-care among these men^(34)^. The aforementioned associations may be related to the different level of knowledge and organization of the health system between genders. These results reinforce the need to pay attention to this differentiation, in order to adjust the management of men and women with 2DM.

Another association identified is related to people with a partner, as they were more sensitized in activating self-care, setting goals and carrying out consultations with specialists. This result allows us to infer that the support received by these people can facilitate self-care and favor a better assessment of the quality of care. This result is consistent with a study carried out in China, with elderly people with 2DM, which identified an association between social support and effective self-management^(35)^. These results reinforce the need for FHS team professionals to involve their partners and/or family members in their daily lives in the care plan, with a view to increasing engagement in treatment.

### Study limitations

Some possible limitations of this study are related to the fact that data collection took place in a period when health care in the municipality was compromised, since, in addition to the reconfiguration of some coverage areas, there was a decrease in the number of teams in the programmatic area, limitation and /or lack of supplies and medications, in addition to frequent moments of paralysis of professionals due to salary arrears, which may have compromised the assessment of users. When considering the instrument, according to the literature, the number of points on the response scale can affect the PACIC score in patients with diabetes.

### Contributions to nursing, health, or public policy

The findings can expand the scope of knowledge, aiming to improve the management of health care aimed at people with 2DM. With regard to care practice, data from this research can guide continuing education policies that qualify professionals from Family Health teams for supported care and patient safety in PHC.

## CONCLUSIONS

People with DM consider that they do not receive individualized treatment, with dialogue and discussion to set goals, and that they are not prepared for self-management of their health condition. They assess that the health care provided by PHC teams is centered on a generalized approach, determined by superficial discussion of care plan, and with gaps in approaching their health habits and preferences.

Thus, it is inferred that care is undervalued and misaligned with the patient safety culture. Still, the little involvement in self-care and the low support on the part of the FHS team in the chronic condition care highlight the need for changes in the care practice for people with DM, since it must be based on active listening, the joint construction of the care plan and team follow-up/support in relation to the agreed goals.
